# Intra-tumor AvidinOX allows efficacy of low dose systemic biotinylated Cetuximab in a model of head and neck cancer

**DOI:** 10.18632/oncotarget.6089

**Published:** 2015-11-07

**Authors:** Loredana Vesci, Ferdinando Maria Milazzo, Anna Maria Anastasi, Fiorella Petronzelli, Caterina Chiapparino, Valeria Carollo, Giuseppe Roscilli, Emanuele Marra, Laura Luberto, Luigi Aurisicchio, Maria Lucrezia Pacello, Luigi Giusto Spagnoli, Rita De Santis

**Affiliations:** ^1^ Biotechnology, Research & Development, Sigma-Tau SpA, 00071 Pomezia, Rome, Italy; ^2^ Tissue Macro Array Lab, University of Tor Vergata, via della Ricerca Scientifica, 00133, Rome, Italy; ^3^ Takis Biotech, Castel Romano, 00128, Rome, Italy

**Keywords:** HNSCC, AvidinOX, Cetuximab, bCet, targeted therapy

## Abstract

For locally advanced and metastatic head and neck squamous cell carcinoma (HNSCC), the current clinical use of Cetuximab in chemo/radiotherapy protocols is often associated to severe systemic toxicity. Here we report *in vitro* data in human FaDu pharynx SCC cells, showing that inactive concentrations of biotinylated Cetuximab (bCet) become active upon anchorage to AvidinOX on the surface of tumor cells. AvidinOX-anchored bCet induces apoptosis and DNA damage as well as specific inhibition of signaling, degradation and abrogation of nuclear translocation of EGFR. In the mouse model of FaDu cancer, we show that intra-tumor injection of AvidinOX allows anti-tumor activity of an otherwise inactive, intraperitoneally delivered, low dose bCet. Consistently with *in vitro* data, *in vivo* tumor inhibition is associated to induction of apoptosis, DNA damage and reduced angiogenesis. AvidinOX is under clinical investigation for delivering radioactive biotin to inoperable tumors (ClinicalTrials.gov NCT02053324) and present data support its use for the local treatment of HNSCC in combination with systemic administration of low dose bCet.

## INTRODUCTION

Head and neck cancer accounts for more than 550,000 cases annually worldwide [1]. The median overall survival for recurrent or metastatic head and neck squamous cell carcinoma (HNSCC) remains less than one year despite a wide armamentarium of therapeutic approaches including anti-EGFR antibody Cetuximab [2, 3]. The administration of Cetuximab in combination with radiotherapy and chemotherapy has shown modest survival improvement in patients with locally advanced and relapsed/metastatic cancer [3, 4] and such improvement is at expenses of increased local and systemic toxicities that deserve consideration and timely management [5]. Therefore, there is a high medical need for improving the cost/benefit ratio of current HNSCC treatments.

We recently described that anchoring biotinylated Cetuximab (bCet) on the surface of AvidinOX-conjugated lung cancer cells, leads to an increase of *in vitro* anti-tumor activity that translates into *in vivo* anti-tumor efficacy of very low doses of nebulized bCet, when administered after nebulized AvidinOX [6]. During the last five years, we reported in several studies that injected AvidinOX exhibits the distinctive property to form Schiff's bases with tissue proteins thus constituting a stable receptor for biotinylated therapeutics that might be very diverse in nature like radiolabeled biotin or biotinylated stem cells [6–9]. Consistently with data obtained with lung cancer cells, here we show anti-tumor activity of AvidinOX-anchored bCet against FaDu pharynx squamous cell carcinoma cells *in vitro*. *In vivo*, we describe that the injection of AvidinOX into FaDu xenografts enables anti-tumor activity of a very low dose bCet, administered intraperitoneally after 24 hours. AvidinOX is currently under investigation in a phase I clinical trial for targeting ^177^Lutetium-biotinDOTA (^177^Lu-ST2210) [10] to inoperable liver metastases (ClinicalTrials.gov NCT02053324). Present results support intra-tumor injection of AvidinOX for targeting systemic low dose bCet in head and neck cancer patients with the expectation to reduce Cetuximab-related toxicity while possibly increasing local anti-tumor efficacy.

## RESULTS

### Biochemical properties of biotinylated cetuximab

**Figure 1:**
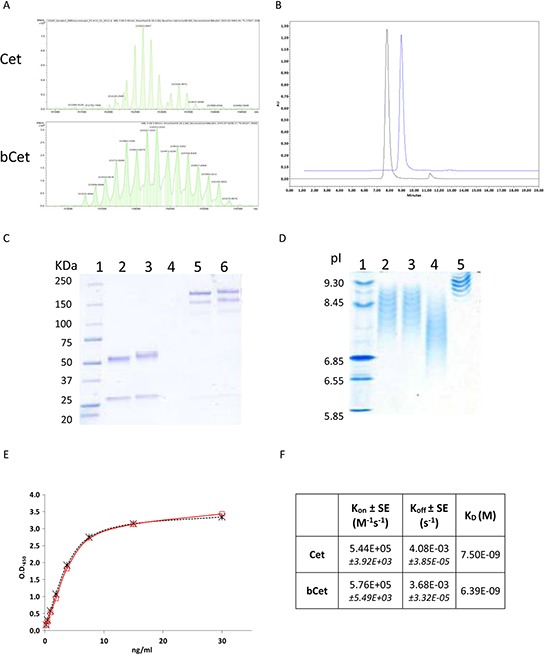
Characterization of biotinylated Cetuximab **A.** Electrospray Ionization Mass Spectrometry profiles of Cetuximab (upper panel) and representative batch of bCet with 4.3 biotins/mole (lower panel). **B.** Size exclusion chromatography of Cetuximab (black line) and bCet (batch as in A, blue line). **C.** SDS-PAGE analysis of Cetuximab and bCet (batch as in A), under reducing (lanes 2 and 3, respectively) and non-reducing (lanes 5 and 6, respectively) conditions. Molecular weight standard in lane 1. **D.** IEF analysis of three batches of bCet with an average of 4.3, 4.4 and 5.7 biotins/mole (lane 2, 3 and 4, respectively). Cetuximab and IEF standards in lane 5 and 1, respectively. **E.** EGFR-specific ELISA of Cetuximab (red line) and bCet (batch as in A, dotted black line). **F.** Cet and bCet kinetic constants by SPR.

Determination of the number of biotin molecules coupled to Cetuximab was performed by Electrospray Ionization Mass Spectrometry (ESI MS). The highest peak of the non-biotinylated Cetuximab exhibited an estimated mass of 152653 Da while the highest peak of bCet exhibited an estimated mass of 154607 Da with a mass difference of 1954 Da. Since biotinylation adds 452.24 Da for each biotin, bCet was calculated to have, in the most represented form, an average of 4.3 biotins/Ig molecule (Figure 1A). Mass spectrometry analysis also identified preferential biotinylation sites on lysine 49, 126, 145, 188, 190 and 207 and lysine 5, 43, 75, 224, 248, 290, 319, 322, 328, 336, 362 and 416 in light and heavy antibody chains, respectively. Molecular integrity of bCet was confirmed by size exclusion chromatography and SDS-PAGE analyses (Figures 1B and 1C, respectively). Isoelectrofocusing analysis showed for bCet batches, lower isoelectric points (8.45–6.85) compared to Cetuximab (9.3–8.65) consequent to the biotinylation of positively charged lysines (Figure 1D). Cytofluorimetry analyses on EGFR^+^ and EGFR^−^ tumor cells confirmed the binding specificity. In fact, bCet did not bind to EGFR^−^ cells as Cetuximab, unless prior cell conjugation with AvidinOX (Supplementary Figure S1). EGFR-specific ELISA showed similar binding potency for bCet and Cetuximab (Figure 1E) and surface plasmon resonance (SPR) indicated similar affinity (Figure 1F), that is consistent with previously published data [11]. Overall results indicated substantial biochemical and biological similarity of bCet with Cetuximab.

An experimental system to test the hypothesis that AvidinOX-bound bCet (potentially tetrameric) might exhibit higher avidity for EGFR compared to the monomeric one, was attempted by SPR. Taking into account all limitation deriving from comparing two different experimental conditions, results showed faster dissociation of EGFR from AvidinOX-bound bCet compared to bCet (Supplementary Figure S2).

### AvidinOX-anchored bCet prevents internalization and induces degradation of EGFR

**Figure 2:**
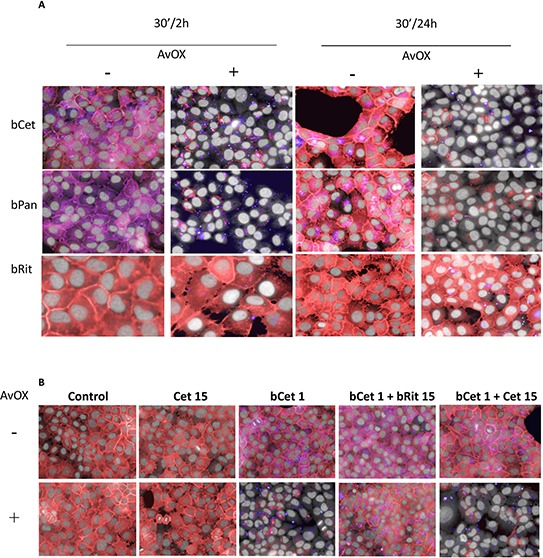
AvidinOX-anchored bCet and bPan induce degradation of EGFR **A.** Fluorescence imaging by High Content Screening (HCS) Operetta of FaDu cells, with and without AvidinOX conjugation and 30-minute incubation with 1 μg/mL CF488-labeled biotinylated Mabs (blue). After 2 or 24-hour cultivation, cells were washed, fixed and stained for the detection of EGFR by AF555-labeled anti-EGFR Mab (red). Draq5 dye staining of nucleus and cytoplasm (grey). Violet is the result of the blue and red dye co-localization. **B.** Cells, with and without AvidinOX conjugation were incubated with medium (Control), 15 μg/mL Cetuximab (Cet) or 1 μg/mL bCet alone or in the presence of 15 μg/mL bRit or Cet. All panels: representative picture of at least 5 fields of triplicate wells. Magnification 60X.

Internalization of EGFR/ligand (EGF or anti-EGFR antibodies) is a physiological mechanism affecting the tumor cell response to growth and inhibition stimuli. To test the effect of AvidinOX anchorage of bCet on EGFR trafficking, FaDu cells were analyzed by High Content Screening (HCS) fluorescence imaging. The presence of FcR on FaDu cells was preliminarily ruled out by cytofluorimetry (data not shown). Biotinylated Panitumumab (human IgG2 anti-EGFR) (bPan) and Rituximab (chimeric IgG1 anti-CD20 Mab) (bRit) were included in the experiments representing a second EGFR-specific and a negative control Mab, respectively. EGF-induced EGFR endocytosis in FaDu cells was proven not to be affected by AvidinOX conjugation (Supplementary Figure S3). HCS was then used to investigate the fate of AvidinOX-anchored biotinylated antibodies. As expected, after 30 minutes, fluorescent bCet but not bRit was found within the cytoplasm of FaDu cells while, in AvidinOX-conjugated cells, both bCet and bRit appeared in dots on the cell surface indicating that bCet internalization was prevented by AvidinOX anchorage (Supplementary Figure S4). Same results were obtained with bPan (not shown). Consistently with previous data in lung cancer cells, where AvidinOX-anchored bCet and bPan induced a dramatic reduction of EGFR consequent to its massive localization within the lysosomes [6], HCS imaging showed that, upon 30-minute incubation with AvidinOX-anchored bCet or bPan but not bRit, EGFR disappeared in FaDu cells (Figure 2A). The degree of the effect was substantial and similar after 2 or 24 hours thus indicating persistence and irreversibility of the effect upon antibody removal. Co-localization of EGFR with lysosomes in Supplementary Figure S5. Interestingly, while EGFR degradation was induced by 1 μg/mL bCet or bPan in AvidinOX-treated cells, Cetuximab and Panitumumab did not affect EGFR in FaDu cells at concentrations up to 200 μg/mL, with or without AvidinOX (Supplementary Figure S6A–S6C). EGFR degradation proved to be strictly dependent on the anchorage of biotinylated anti-EGFR antibodies to AvidinOX. In fact, no degradation occurred when bCet binding to AvidinOX was competed by an excess of unrelated biotinylated antibody (bRit) while, it persisted in the presence of an excess of Cetuximab, despite the expected competition on EGFR binding (Figure 2B). Same results were obtained with bPan (not shown).

### AvidinOX-anchored bCet inhibits phosphorylation and signaling of EGFR

**Figure 3:**
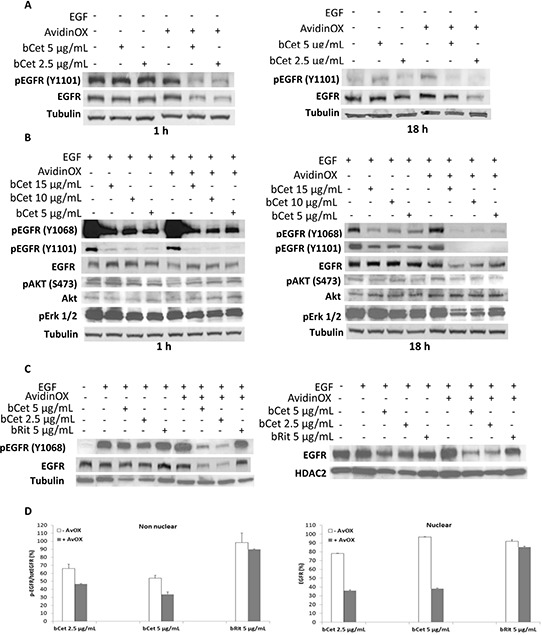
AvidinOX-anchored bCet blocks EGFR signaling, induces receptor degradation and prevents nuclear translocation Cells were serum-starved 24 hours and then cultivated, with or without AvidinOX conjugation in culture medium or in the presence of indicated antibodies. Whole cell lysates or sub-cellular fractions were prepared at different times following treatment and then subjected to Western blotting or ELISA analyses for indicated target proteins. **A.** Whole cell lysates. **B.** Whole cell lysates from EGF-stimulated cells. **C.** Nuclear (right panel) and non-nuclear (left panel) fractions of EGF-stimulated cells, 18 hours after treatment. **D.** ELISA titration of phosphorylated and total EGFR in non-nuclear (left panel) and nuclear (right panel) fractions of EGF-stimulated cells, as in C. Data are expressed as % residual pEGFR/EGFR (non-nuclear) or nuclear EGFR compared to cells in culture medium. Error bars: mean of duplicates ± SD. EGF was added 30 minutes before cell lysis, where indicated. All panels: representative data from at least two independent experiments.

To ascertain whether AvidinOX-anchored bCet would have been able to block EGFR signaling in FaDu cells, cultures were treated with bCet with and without EGF stimulation. EGFR phosphorylation and downstream signaling were assessed by Western blotting. As shown in Figure 3A, treatment with bCet significantly reduced basal level of phosphorylated EGFR (pEGFR) in AvidinOX pretreated cells and caused a dramatic reduction of total EGFR. Both effects were observed after 1 hour and persisted at least up to 18 hours. In EGF-stimulated cells, treatment with bCet was effective in reducing EGFR phosphorylation at both 1068 and 1101 tyrosine residues with a more pronounced effect in AvidinOX pretreated cells, where the effect was also accompanied by a strong reduction of total EGFR and by decreased level of phosphorylated-Akt and Erk 1/2, which are known to be downstream effectors of EGFR signaling (Figure 3B). Western blotting data showing a dramatic reduction of EGFR are consistent with HCS imaging data in Figure 2 and Figure S6A–S6C. We previously showed, in several lung cancer cell lines, that AvidinOX-anchored bCet induces a dramatic decrease of total and activated EGFR in both nuclear and non-nuclear cell compartments [6]. As shown in Figure 3C, a very strong reduction of phosphorylated non-nuclear (left panel) and nuclear (right panel) EGFR was observed in EGF-induced FaDu cells upon AvidinOX anchorage of bCet but not bRit. These data were further confirmed by ELISA titration of EGFR on the same cellular extracts (Figure 3D).

### AvidinOX-anchored bCet induces apoptosis and interferes with pro-angiogenic pathways

**Figure 4:**
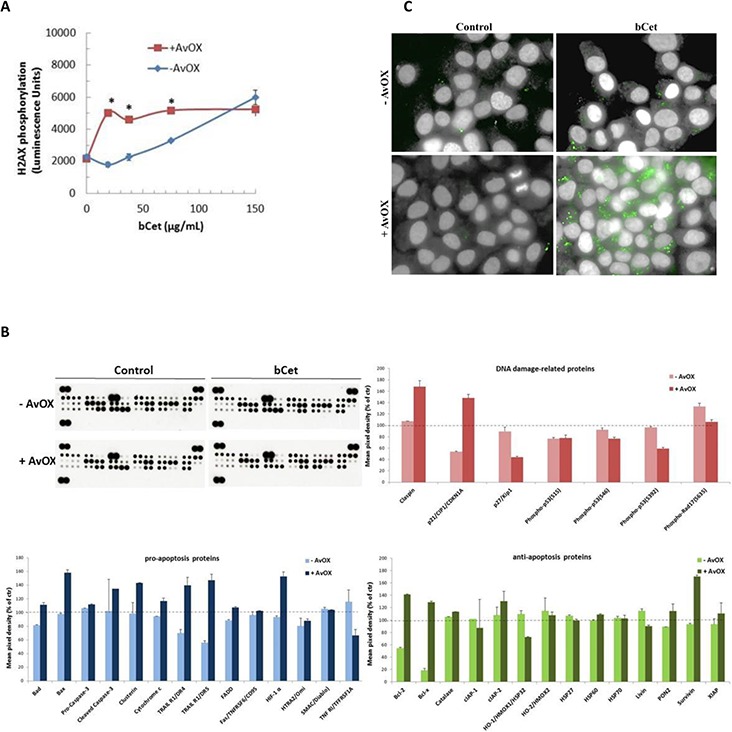
AvidinOX-anchored bCet induces H2A.X phosphorylation and apoptosis **A.** FaDu cells, with and without AvidinOX conjugation, were treated 4 hours with medium or bCet. Histone H2A.X phosphorylation was measured by using a Phosphorylation Chemiluminescence Detection kit. Each value represents the mean ± SE of two independent experiments. (*p < 0.05 vs cells without AvidinOX, Mann-Whitney's test). **B.** Array membrane representative image of FaDu cells, with and without AvidinOX conjugation, treated 48 hours with medium (Control) or 5 μg/mL bCet. EGF induction the last 30 minutes. Apoptosis-related proteins were measured on whole cell lysates using the Human Apoptosis Array kit. Spot pixel densities were recorded and analyzed by using image analysis software. Average background signal was subtracted from each spot and the normalized mean pixel densities were plotted according to target family. Data (mean of duplicates ± SD) were expressed as percentage with respect to baseline values (100%) of control cells (with and without AvidinOX). **C.** HCS analysis of FaDu cells, 48 hours after 1-hour contact with medium (Control) or 5 μg/mL bCet, with and without AvidinOX conjugation. After treatment, cells were washed, fixed and stained for the detection of cleaved-caspase-3 (green). Draq5 dye staining of nucleus and cytoplasm (grey). Each panel, representative picture of at least 5 fields of triplicate wells. Magnification 60X. Note: in all experiments, AvidinOX conjugation did not affect baseline values.

Several *in vitro* experiments were performed with FaDu cells for testing the effect of AvidinOX-anchored bCet on molecular pathways. As shown in Figure 4A, AvidinOX-anchored bCet was about 10 times more effective than bCet at inducing phosphorylation of the early marker of DNA damage, histone H2A.X (γH2A.X), after 4-hour incubation. To ascertain whether the prolonged treatment with AvidinOX-anchored bCet could also result in induction of apoptosis or other cell death-related pathways, FaDu cells, with or without AvidinOX pretreatment, were cultivated 48 hours in the presence of bCet and then whole cell lysates were analyzed for multiple protein expression by using a Proteome Profiler Array (Figure 4B, upper left panel). Results after densitometric analysis showed that AvidinOX-anchored bCet caused an increase of several pro-apoptotic proteins associated to both extrinsic and intrinsic apoptotic pathways (i.e. TRAILR1/DR4 and TRAILR1/DR5, Bax, cleaved caspase-3, clusterin, cytochrome C) (Figure 4B, lower left panel). Interestingly, the treatment with bCet, without AvidinOX, was associated to a strong down-regulation of anti-apoptotic effectors belonging to the Bcl-2 protein family (Figure 4B, lower right panel). Activation of P53 was not observed with any treatment most likely because this is an event occurring at earlier time points. Nevertheless, AvidinOX-anchored bCet caused up-regulation of the cell cycle inhibitor p21^Cip1^ and stress-associated replication regulator protein Claspin (Figure 4B, upper right panel). In agreement with array protein expression data, higher cleavage of caspase-3 and up-regulation of p21^Cip1^ in the same FaDu cell cultures, were observed with AvidinOX-anchored bCet, compared to bCet, by HCS analysis (Figure 4C and Supplementary Figure S7, respectively).

**Figure 5:**
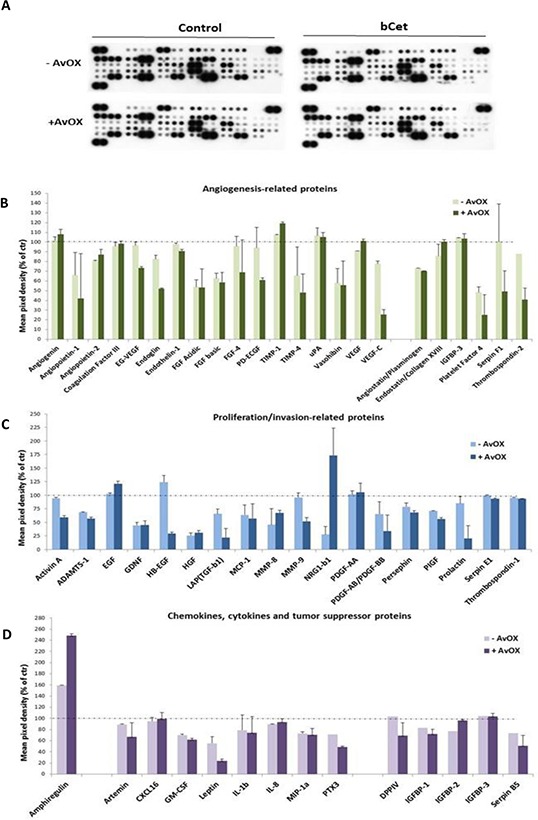
AvidinOX-anchored bCet affects pro-angiogenic and pro-tumorigenic pathways FaDu cells, with and without AvidinOX conjugation, treated 72 hours with medium (Control) or 5 μg/mL bCet, with EGF induction the last 30 minutes. Analysis of secreted angiogenesis- and proliferation-related proteins was carried out using the Proteome Profiler Array kit. Spot pixel densities were collected and analyzed using image analysis software. Average background signal was subtracted from each spot and the normalized mean pixel densities were plotted according to target family. Data (mean of duplicates ± SD) were expressed as percentage with respect to baseline values (100%) of control cells (with and without AvidinOX). **A.** Array membrane representative image. **B.** Angiogenesis-related proteins. **C.** Proliferation/invasion-related proteins. **D.** Chemokines and cytokines. Note: in all experiments, AvidinOX conjugation did not affect baseline values.

In order to assess whether treatment with bCet could also induce molecular modulation associated with angiogenesis, tumor growth, invasion and metastasis, the level of specific proteins secreted in the conditioned culture medium of FaDu cells was measured by using a specific Proteome Array kit (Figure 5A). As shown in Figures 5B and 5C, AvidinOX-anchored bCet was more effective than bCet decreasing secretion of several pro-angiogenic factors, as well as many growth factors and invasion-associated enzymes. Noteworthy, higher reduction of secreted chemokines having pro-tumorigenic/invasion effects was observed with anchored-bCet compared to bCet paralleled by an impressive increase of secreted Amphiregulin which is known to be a relevant predictive marker of tumor cell sensitivity to Cetuximab [12] (Figure 5D).

### Intra-tumor injection of AvidinOX enables anti-tumor efficacy of low dose systemic bCet

**Figure 6:**
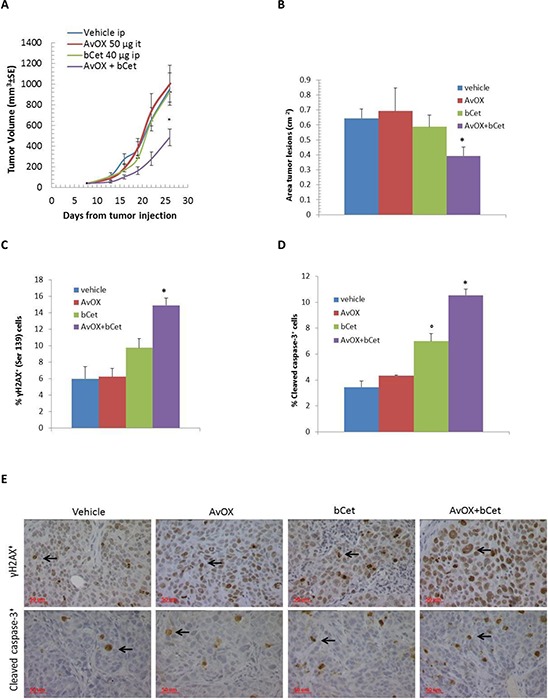
Intra-tumor injection of AvidinOX allows anti-tumor efficacy of low dose intraperitoneal bCet **A.** Tumor volume of FaDu s.c. xenotransplants in mice treated i.p. with 40 μg bCet, 8, 15 and 22 days after tumor cell transplantation, with or without (vehicle) injection of AvidinOX (50 μg), administered intra-tumor (tumor masses 40–50 mg) 24 hours before each bCet dose. Data are mean ± SE of 8 mice per group. **B.** Average area of tumor masses measured on hematoxylin-eosin stained paraffin-embedded sections of mice in A. **C.** Percentage of tumor cells (mean ± SE) immunostained for phosphorylated histone H2A.X (γH2A.X) in tumor sections of mice in A (5 fields from two serial sections/mouse). **D.** Percentage of tumor cells (mean ± SE) immunostained for cleaved caspase-3 in tumor sections of mice in A (5 fields from two serial sections/mouse). **E.** Representative pictures of data in C and D (Magnification 40X). Panels A–D. Mann-Whitney U test *p < 0.05 versus bCet. Data from one representative study out of two.

The half-life of injected AvidinOX into normal tissues and tumor masses was previously shown to be about 14 and 7 days, respectively [7, 13]. In a preliminary dose-finding study, 40 μg/mouse bCet, administered i.p. three times once/week to mice bearing subcutaneous xenografts of FaDu cells, was found to be a suboptimal treatment (Supplementary Figure S8). Therefore, mice with FaDu xenografts were treated with this bCet dose with and without intra-tumor pre-treatment with AvidinOX, 24 hours before each bCet administration. As shown in Figure 6A, significant tumor growth inhibition was only observed in AvidinOX-treated tumors, with a tumor volume inhibition of 56%. Analysis of tumor sections by histology confirmed significant reduction of neoplastic areas in this group (Figure 6B), paralleled by higher phosphorylation of histone H2A.X (γH2A.X) and increased level of cleaved caspase-3, as determined by immunohistochemistry (Figures 6C, 6D, 6E).

**Figure 7:**
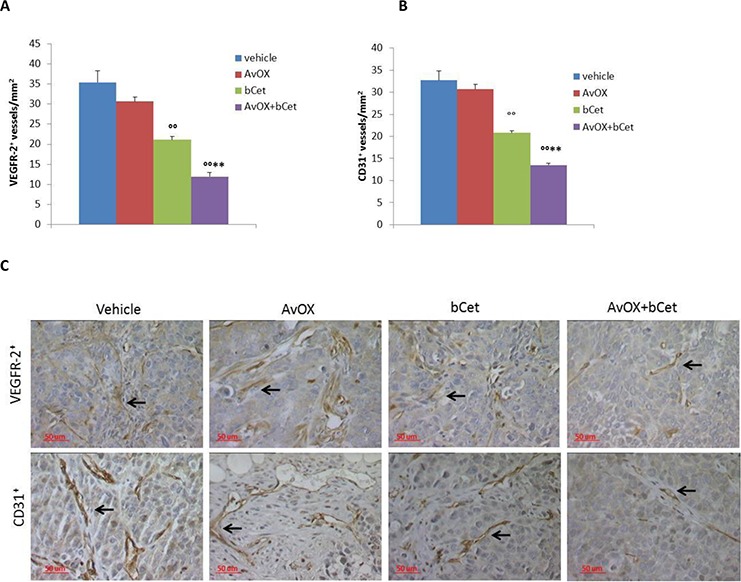
Anti-tumor efficacy of low dose intraperitoneal bCet in AvidinOX-treated tumors correlates with inhibition of angiogenesis **A.** VEGFR-2 and **B.** CD31 positive vessels by immunohistochemistry in human FaDu tumor xenografts from mice treated i.p. with bCet with or without previous intra-tumor injection of AvidinOX of study in Figure 6. Microvessel density (MVD) is expressed as mean number of vessels per square millimeter ± SE of tumor samples from each group (12 selected fields from two serial sections/mouse).°°*p* < 0.01 vs vehicle and **p < 0.01 vs bCet by Mann-Whitney U test. **C.** Representative immunohistochemistry pictures of data in A and B. Magnification 40X.

Additional serial sections from the same tumor xenografts were used to investigate angiogenesis. Microvessel density (MVD) was evaluated by counting the number of VEGFR2^+^ and CD31^+^ vessels. Results showed that the anti-angiogenic activity of bCet was significantly enhanced upon AvidinOX anchorage (Figures 7A, 7B, 7C). No toxicity was observed in all experimental groups as indicated by lack of body weight loss (data not shown). Overall, *in vivo* data proved to be consistent with *in vitro* results obtained with FaDu cells.

## DISCUSSION

The anti-EGFR monoclonal antibody Cetuximab is approved in Europe and US for use in combination with chemo/radiation in patients with locally advanced and as monotherapy for recurrent and metastatic HNSCC [14–17]. Unfortunately, modest anti-tumor efficacy, together with significant side effects and costs remain major obstacles to its clinical wide spreading [4, 18]. The dose of intravenous Cetuximab can be as high as 2 g/month and treatment can go on for several months. The British health system found the addition of Cetuximab to radiotherapy to be cost-effective compared to single modality radiotherapy while Cetuximab with chemotherapy is not recommended. Whereas, in the US, where economic evaluations are not incorporated in the drug approval process, addition to chemotherapy is the preferred regimen [19]. To assess the cost effectiveness of adding Cetuximab to chemotherapy in patients with recurrent or metastatic HNSCC, the Canadian public healthcare system adopted a model to project the lifetime clinical and economic consequences of the disease in a three year time horizon. In the base case, addition of Cetuximab to platinum-based chemotherapy led to an increase in cost of $36,000 per person, resulting in an incremental cost effectiveness ratio (ICER) of $386,000 per quality-adjusted life-year (QALY) gained and concluding that Cetuximab could only be economically attractive if the cost of Cetuximab is substantially reduced or if future research can identify predictive markers to select patients most likely to benefit from the addition of Cetuximab to chemotherapy [20]. It is known that Cetuximab inhibits oral squamous cell carcinoma invasion and metastasis directly via degradation of epidermal growth factor receptor [21] and indirectly via inhibition of neo-angiogenesis [22]. Cetuximab can also exert pro-apoptotic activity particularly when used in combined therapies [23, 24]. With regard to DNA damage in response to irradiation, Cetuximab has been reported to inhibit EGFR translocation to the nucleus that is known to activate major DNA repair pathways [25]. In contrast with this observation, others indicate that intra-cellular trafficking of EGFR is enhanced by Cetuximab [26] and there is a wide literature correlating poor response to Cetuximab with EGFR nuclear localization thus making nuclear EGFR an important molecular target in cancer [27–29]. We recently reported that AvidinOX-anchored bCet inhibits dimerization and signalling, blocks endocytosis, induces massive lysosomal degradation and abrogates nuclear translocation of EGFR, in lung cancer cells at doses otherwise not effective. This translates into utility of nebulizing tiny doses of bCet that, when delivered after nebulized AvidinOX, exert therapeutic effect in lung cancer models [6]. Consistently with previous data in lung cancer, here we show that intra-tumor injection of AvidinOX turns a low systemic dose of bCet into an effective anti-tumor treatment in the FaDu model of head and neck cancer. Growth inhibition of FaDu tumor xenografts is shown to be associated with the induction of apoptosis and DNA damage and marked inhibition of angiogenesis. *In vivo* results are in agreement with *in vitro* data indicating that AvidinOX anchorage turns ineffective bCet concentrations into a treatment leading to massive EGFR degradation and to activation of pro-apoptotic and anti-angiogenesis signals. As with previous lung cancer data, the second anti-EGFR biotinylated antibody bPan behaves similarly as bCet in FaDu, suggesting that engagement of EGFR by any AvidinOX-anchored anti-EGFR antibody might lead to the same outcome. Previous data had clearly demonstrated that potency improvement of biotinylated anti-EGFR antibodies was dependent on the interaction of biotin with AvidinOX. In fact, the non-biotinylated controls, at suboptimal doses, were less or not effective with or without AvidinOX [6]. Presently, we show in Supplementary Figure 6A–6C that Cetuximab and Panitumumab fail to induce EGFR degradation in FaDu cells, at dose as high as 200 μg/mL, with or without AvidinOX. We also prove that AvidinOX anchorage is essential to bCet and bPan activity as competition on AvidinOX occupancy by a not-relevant biotinylated antibody prevents their anti-EGFR activity. Overall data confirm that AvidinOX anchorage endows anti-EGFR antibodies with peculiar properties, the most evident of which is the induction of dramatic degradation of EGFR. Present data support the clinical use of intra-tumor AvidinOX to enable therapeutic efficacy of low doses anti-EGFR antibodies in HNSCC patients. Dose reduction of these antibodies is expected to have beneficial effects on both overall tolerability and cost of treatment. Regarding tolerability, AvidinOX has been proven to be very well tolerated either when injected in tissues or when inhaled by both rodents and non-human primates, despite its known immunogenicity [6–9, 13, 30–33]. Very good tolerability and no treatment-related adverse events have been recorded in eight patients, with inoperable liver metastasis, who were treated to date with intra-tumor AvidinOX and intravenous ^177^Lu-ST2210 (NCT02053324). Regarding cost of the treatment, this is expected to be substantially lower than current Cetuximab treatments because of the low amount of the expensive antibody to be used and low manufacturing costs of Avidin, which is extracted from egg white and oxidized by quite simple biochemical processes [13]. Present results in HNSCC, together with previous results in lung cancer models, point to AvidinOX as a wide applicable tool for making low doses of anti-EGFR antibodies effective against cancer. Ongoing studies in our laboratories show that the principle is also valid for anti-ErbB2 and ErbB3 antibodies (manuscript in preparation). The latter results, although not obvious, are somehow expected based on the biological similarities of the targeted receptors, all belonging to the EGFR family [34]. On the other hand, the use of AvidinOX for targeting antibodies to receptors of different families, to other surface molecules or to soluble factors is still speculative but very exciting if we think to the possibility to reduce the doses of expensive and frequently toxic reagents. Particularly, antibodies against the immune check points inhibitors, that are currently becoming very popular because of their proven efficacy [35, 36] might find benefit from an AvidinOX-based targeted delivery that could allow reduction of doses and improvement of tolerability.

Interestingly, AvidinOX would easily allow the targeting of combination therapies to tumors, as well as to other diseased tissues, with therapeutics that might include a variety of biotinylated agents spanning from radioactive biotin, as in the current clinical trial, to biotinylated antibodies or biotinylated cells for each of which proof of concepts were previously produced [6–9].

All results collectively taken encourage further use of AvidinOX in additional clinical protocols.

## MATERIALS AND METHODS

### Cells and AvidinOX conjugation

Human squamous cell carcinoma of the pharynx FaDu, human squamous cell carcinoma of the tongue OSC-19 and human melanoma SKMel28 cells were obtained from the American Type Culture Collection (ATCC) and maintained in a humidified atmosphere with 5% CO_2_ at 37°C. Working cell banks were established and all experiments were performed using cells within 6–8 passages after thawing.

AvidinOX^®^ (registered brand of Sigma Tau) was prepared by Areta International (Varese, Italy) as a lyophilized form, according to previously described methods [13]. After reconstitution with water for injection, the protein was 3.0 mg/mL, in acetate buffer pH 5.2 with mannitol and NaCl. For AvidinOX conjugation, pellets of 5×10^5^ cells or adherent cells (96- or 24-well plates) were washed with PBS and incubated 1 hour at 4°C with 100 μL of 100 μg/mL AvidinOX in PBS. Cells were then washed with DMEM and used for further *in vitro* experiments.

### Preparation and characterization of biotinylated antibodies

For biotinylation of Cetuximab (Erbitux^®^; Merck Serono), Panitumumab (Vectibix^®^; Amgen) and Rituximab (MabThera^®^; Roche), 100 mg of antibody were subjected to buffer exchange by ultrafiltration on Amicon Ultra 30K (Millipore) and brought to a concentration of about 10 mg/mL in PBS. Activated 2X-AHbiotin-N-Hydroxysuccinimide ester (ST3297, Sigma-Tau) was added at 1:10 Mab:biotin molar ratio. The reaction mixture was incubated 2 hours at room temperature under mild shaking, loaded on SEC disposable PD-10 column (G-25, Amersham-Pharmacia) and eluted with PBS. Biotinylated antibody fractions were pooled based on OD_280_ values and finally filter sterilized. Endotoxins were tested by LAL test (Endosafe PTS/MCS Cartridges, Charles River).

The biotinylation level was assessed by HABA test [37] and/or by Electrospray Ionization Mass Spectrometry (ESI MS). For ESI MS, the samples were pretreated with 8M Urea and then sprayed through a capillary at high voltage into the MS instrument where the mass over charge ratio (m/z) was measured. The mass of the molecule was determined using a deconvolution algorithm (MaxEnt Software). Positions of biotinylated lysines were analyzed by nano-LC-MS/MS peptide sequencing (Alphalyse) and protein identification was based on a probability-scoring algorithm (http://www.matrixscience.com). Briefly, the protein samples were reduced and alkylated with iodoacetamide, i.e. carbamidomethylated, and subsequently digested with trypsin. The resulting peptides were concentrated by Speed Vac lyophilization and redissolved for injection on a Dionex nano-LC system and MS/MS analysis on a Bruker Maxis Impact QTOF instrument. The MS/MS spectra were used for UniProt and NCBI database searching by using the Mascot, version 2.4, software.

Purity and integrity were confirmed by SEC-HPLC. Antibodies (300 μg in 30 μL) were loaded on a TSKgel G3000 SWXL (7.8 × 300 mm) column (Tosoh Bioscience). Mobile phase: 100 mM phosphate buffer solution (pH 7.0), 300 mM NaCl/Acetonitrile (90:10). Flow rate: 1 mL/min. Detection by 280 nm absorbance. SDS-PAGE and IEF analyses were performed according to standard methods [38, 39]. For electrophoresis, samples were separated on a linear 4–15% acrylamide gradient gel and stained with Coomassie Brilliant Blue R250. For IEF, the analysis was performed in native conditions and samples (5 μg/lane) separated on IEF pH 3–9 gel containing 5% polyacrylamide and stained with Blue Bandit.

Immunoreactivity of biotinylated Cetuximab (bCet) and biotinylated Panitumumab (bPan) was tested by antigen-specific ELISA. Briefly, Immuno MAXISORP 96-well plates (Nunc) were coated overnight at 4°C with 50 ng/well of recombinant human EGF-R/Erb1 Fc chimera (R&D). Plates were washed with PBS 0.1% Tween-20 (PBS-T), blocked 2 hours with PBS-T, 1% BSA, and incubated with serial dilutions of antibodies 1 hour at room temperature (RT). After washings, horseradish peroxidase (HRP)-conjugated anti-human K light chain antibody (Sigma Aldrich), diluted 1:1000 in blocking solution, was added 1 hour at RT. After three washings, 200 μL/well TMB substrate (Sigma Aldrich) were added and after 30 minutes at RT, the reaction was blocked by H_2_SO_4_ solution, and optical density at 450 nm was measured by ELISA reader (Tecan).

Specificity of biotinylated antibodies was tested by cytofluorimetry. Pellets of FaDu, OSC-19 or SKMel28 cells, with and without AvidinOX-conjugation, were incubated 1 hour at 4°C with antibodies and their related biotinylated derivatives. After washings, cells were incubated with mouse PE-conjugated anti-human Ig (BD). Analysis was carried out through FACScalibur (BD).

Affinity analysis by Surface Plasmonic Resonance (SPR) was carried out by Biacore T200 instrument (GE). The binding kinetics of Cetuximab and bCet to recombinant EGFR-Fc chimera (R&D) immobilized by standard amino coupling procedure to a CM5 sensor chip were measured by injecting the two antibodies in the 250–15.6 nM concentration range (duplicates). Evaluation of k_on_ and k_off_ rate constants was performed by BIA Evaluation software version 3.2, using the bivalent analyte fitting model and K_D_ calculated as the ratio of the two constants.

### H2A.X phosphorylation (γH2A.X) assay

Tumor cells were plated onto 96-well clear bottom black plates (Corning) in culture medium supplemented with 10% FBS. Twenty-four hours later, cells were washed with DPBS and were treated with AvidinOX (100 μg/mL) 30 minutes at 37°C. After removal of AvidinOX solution, the cells were washed and treated 4 hours with biotinylated Cetuximab in culture medium containing 2% FBS. Histone H2A.X phosphorylation at serine 139 was measured by ELISA using the H2A.X Phosphorylation Chemiluminescence Detection kit (Upstate), according to the manufacturer's instructions.

### EGFR signaling by western blotting

FaDu cells were seeded in 10-cm culture plates (3×10^6^ cells/plate) in EMEM 10% FBS, incubated overnight at 37°C and then starved 24 hours in serum-free medium. Cells, with and without AvidinOX (100 μg/mL) conjugation, were then cultivated for different times with biotinylated antibodies at various concentrations. In some experiments, antibodies were removed after 1 hour contact and then cells were cultivated 18 hours in serum-free medium. EGFR activation was performed by adding 100 ng/mL EGF (R&D) 30 minutes before cell lysis. At the end of culture, cells were washed twice with ice-cold PBS and then whole cell lysate was prepared by incubation, 10 minutes in ice, with lysis buffer (Cell Signaling) supplemented with protease and phosphatase inhibitors. Cell lysates were subjected to sonication prior to centrifugation at 14,000 × *g*, 10 minutes at 4°C, to remove cell debris. In some experiments, cytosolic/membrane fractions and nuclear extracts were prepared and analyzed as previously described [6]. Protein content was determined by Bradford method. Equal amounts of soluble proteins were separated on SDS-PAGE and then transferred to nitrocellulose membrane (Amersham Hybond-ECL, GE). Membranes were blocked 3 hours at room temperature with 5% non-fat dry milk in PBS, 0.05% Tween-20 (PBS-T) before overnight incubation, at 4°C, with one of the following primary antibodies: pEGFR (Tyr1068) (#2236), EGFR (#4267), pAKT (#4058), AKT (#9272) and pERK 1/2 (#9101) from Cell Signaling; pEGFR (Tyr1101) (#ab76195) from abcam. Immunoblotting with anti-α-tubulin (#T5168, Sigma Aldrich) or anti-HDAC2 (#2540, Cell Signaling) antibodies was done to confirm equal protein loading for non-nuclear/whole cell lysate and nuclear protein extracts, respectively. After washings with PBS-T, membranes were incubated 1 hour with the appropriate secondary HRP-conjugated anti-rabbit (1:3000) or anti-mouse (1:2000) IgG antibody (Sigma Aldrich and Amersham GE, respectively). Immunoreactive bands were visualized by enhanced chemiluminescence detection (Amersham ECL plus, GE) and analyzed by phosphoimaging (STORM, Molecular Dynamics) or by exposure to X-ray film (Amersham Hyperfilm ECL, GE). Densitometric analysis was performed using an image analysis software (ImageQuant TL Image Analysis Software v8.1, GE).

### EGFR expression assay

FaDu cells were seeded in 10-cm culture plates, starved 24 hours in serum-free medium and then, with or without AvidinOX conjugation, incubated 1 hour with biotinylated antibodies. After washing, cells were cultivated 18 hours in serum-free medium, with EGF induction the last 30 minutes (100 ng/mL). Phosphorylated and total EGFR in nuclear and non-nuclear protein fractions were assessed by specific PathScan Sandwich ELISA kits (Cell Signaling), according to manufacturer's instructions.

### Apoptosis assay

FaDu cells were seeded in 10-cm culture plates, starved 24 hours in serum-free medium and then, with and without AvidinOX conjugation, cultivated 48 hours in the presence or absence of bCet 5 μg/mL, with EGF induction the last 30 minutes (100 ng/mL). Whole cell lysates were then prepared and used for assessing the expression of various proteins involved in apoptosis and DNA repair, through the Human Apoptosis Array kit (R&D), according to manufacturer's instructions.

After exposition of membranes to X-ray film, pixel densities were collected through the Imager 600RGB (GE) and analyzed by image analysis software (ImageQuant TL Image Analysis Software v8.1, GE). Average background signal was subtracted from each spot and the normalized mean pixel densities plotted according to target family. Data were expressed as percentage with respect to values measured in control cells.

### Measurement of proliferation- and angiogenesis-related proteins in conditioned media

FaDu cells, with and without AvidinOX (100 μg/mL) conjugation, were cultivated in serum-free medium with biotinylated antibodies. At each time point, conditioned culture media (CM) were collected, centrifuged at 400 × *g*, divided into aliquots and stored at −80°C until use. Secretion of angiogenesis- and proliferation-related proteins in CM was assessed using the Proteome Profiler Array assay (R&D), according to manufacturer's instructions.

Acquisition and analysis of pixel densities were performed as described before. Data were expressed as percentage with respect to values measured in CM of control cells.

### High content screening (HCS) fluorescence imaging

FaDu cells were seeded in 96-well microtiter plates (3–6 × 10^3^/well) and cultivated 3 days, starved 24 hours in serum-free medium and then, with or without AvidinOX conjugation (100 μg/mL), incubated 30 minutes with medium or CF488-labelled antibodies. Antibodies were removed by washing with medium and cells analyzed for fluorescence by High Content Screening (HCS) system Operetta (Perkin Elmer), after cultivation in serum-free medium for the indicated time.

EGFR was detected by AF555-conjugated rabbit anti-EGFR antibody (D38B1) (Cell Signaling), added after cell fixation with 4% formaldehyde in PBS, permeabilization with PBS, 0.2%Tween-20 (PBS-T) and blocking with 2% BSA in PBS-T. Cells were counterstained with Draq5 dye (Cell Signaling).

For the detection of cleaved-caspase-3 and P21^Cip1^, FaDu cells with or without AvidinOX conjugation (100 μg/mL) were incubated 1 hour with the biotinylated-antibodies (5 μg/mL) and then, after washing, cultivated 48 hours in serum-free medium, with EGF induction (100 ng/mL) the last 30 minutes. Cells were then washed, fixed and permeabilized. Cleaved-caspase-3 and P21^Cip1^ were detected by using specific rabbit polyclonal antibodies (#9661, Cell Signaling and sc-397, Santa Cruz, respectively) and PE-conjugated goat anti-rabbit Ig as secondary antibody (#554020, BD).

### *In vivo* studies

Animal studies were performed in accordance with the “Directive 2010/63/UE” on the protection of animals used for scientific purposes, made effective in Italy by the Legislative Decree 4 March 2014, n. 26, and ARRIVE guidelines [40]. Female athymic nude mice, 5 to 6 weeks old, were purchased from Harlan Laboratories (Udine, Italy). All procedures performed on the animals were approved by Animal Welfare Body and authorized by the Italian Ministry of Health, 46/2014-PR. At the end of the treatment period and before necropsy, mice were euthanized by CO_2_ asphyxia as indicated in the AVMA (American Veterinary Medical Association) Panel on Euthanasia and according to the guidelines described in UKCCR, United Kingdom Co-ordinating Committee on Cancer Research, 1998.

Mice were maintained in a pathogen-free facility and were inoculated subcutaneously, in the right flank, with 100 μL suspension of 5 × 10^6^ FaDu cells in Hanks' balanced salt solution. Tumor-bearing mice were randomized into groups of 8 and received 50 μg/16.6 μL of AvidinOX intratumorally or the same volume of AvidinOX formulation buffer (vehicle), 24 hours before each intraperitoneal injection of 40 μg/mouse bCet. Treatment started 8 days after tumor transplantation (average size of tumor masses 40–50 mg). Tumor measurements were performed by digital Vernier caliper twice per week and tumor volume (TV) calculated by the equation: volume = length × (width)^2/^2.

Efficacy of treatment was assessed as: TV inhibition percentage (TVI%) in treated versus control mice, calculated as: TVI% = 100 - (mean TV treated/mean TV control x100). When tumors reached a volume of about 1000 mm^3^, mice were euthanized. To examine the possible toxicity of treatment, body weight was recorded throughout the study. Percent of body weight loss (BWL) was calculated as: 100 - (mean BW_dayx_/mean BW_day1_x100), where day 1 is the first day of treatment and day × is any day after (maximum BWL%).

### Histology and immunohistochemistry

Tumors were harvested from mice sacrificed 4 hours after the last antibody administration. Tumor masses were fixed 12 hours at 4°C in 10% phosphate-buffered formalin. Then, they were dehydrated in ascending concentrations of ethanol, cleared with xylene and paraffin embedded. Tissue blocks were cut by using a rotary microtome into 5-μm sections and processed for histology and immunohistochemistry (IHC). For histology, hematoxylin/eosin staining was performed according to standard methods. Area of tumor lesions was calculated by AlexaSoft X-PRO software and data expressed in cm^2^ (40X magnification).

For immunohistochemistry, sections after deparaffination and rehydration were treated 15 minutes with 10 mM citrate buffer and 0.05% Tween 20 (pH 6.0) in a microwave for antigen retrieval, followed by quenching of endogenous peroxidase activity 5 minutes with 3% H_2_O_2_ in PBS. Sections were then incubated with specific antibodies against phospho-Histone H2A.X (Ser139) (#2577) and cleaved caspase-3 (#9664), from Cell Signaling, or against CD31 (#ab28364), from Abcam, diluted in blocking buffer (5% Goat serum and 0.4% Triton X-100, in PBS, for γ-H2A.X and cleaved caspase-3; 10% Goat serum in PBS for CD31) overnight at 4°C in a humidified chamber. Negative controls were incubated without primary antibodies. Sections were incubated with appropriate biotinylated secondary antibody (1:300) and then with the detection solution containing conjugated HRP-streptavidin and 3,3′-diaminobenzidine (ABC kit, Vector). Sections were ultimately counterstained with hematoxylin. Images were taken by microscope (Eclipse E800, Nikon) equipped with a JVC KY-F55B color video digital camera.

For VEGFR-2 evaluation, sections after deparaffination and rehydration were treated 15 minutes with 1 mM EDTA and 0.05% Tween 20 (pH 8.0) in a microwave for antigen retrieval, followed by quenching of endogenous peroxidase activity for 5 minutes with 3% H_2_O_2_ in PBS. Next, sections were incubated with a specific antibody anti-VEGFR-2 (#2479, Cell Signaling), diluted in blocking buffer (5% Goat serum in Tris buffered-saline with 0.05% Tween 20), overnight at 4°C in a humidified chamber.

IHC staining for γ-H2A.X and cleaved caspase-3 was quantified as: number of positive cells (brown cells) × 100/total number of cells, in 5 fields from two serial sections/mouse.

The mean number of VEGFR-2 and CD31 positive vessels per mm^2^ viable tumor was quantified in 24 arbitrary selected fields/tumor.

All tissue sections for immunohistochemical analysis were scored by two independent pathologists using light microscope and subsequently data confirmed by computerized measurements [41].

### Statistical analysis

All data were expressed as a mean of values ± standard error (SE) or ± standard deviation (SD) and differences between groups were analyzed using non-parametric Mann-Withney's U test. Differences were considered significant at *p* ≤ 0.05.

## SUPPLEMENTARY FIGURES


