# Supplementary material for: Intra-tumor AvidinOX allows efficacy of low dose systemic biotinylated Cetuximab in a model of head and neck cancer
Source: Oncotarget. 2015 Nov 7;7(1):914–28. doi: 10.18632/oncotarget.6089 (PMC4808042; doi:10.18632/oncotarget.6089)
Supplement: Supplementary file 1 [file oncotarget-07-0914-s001.pdf]

SUPPLEMENTARY FIGURES

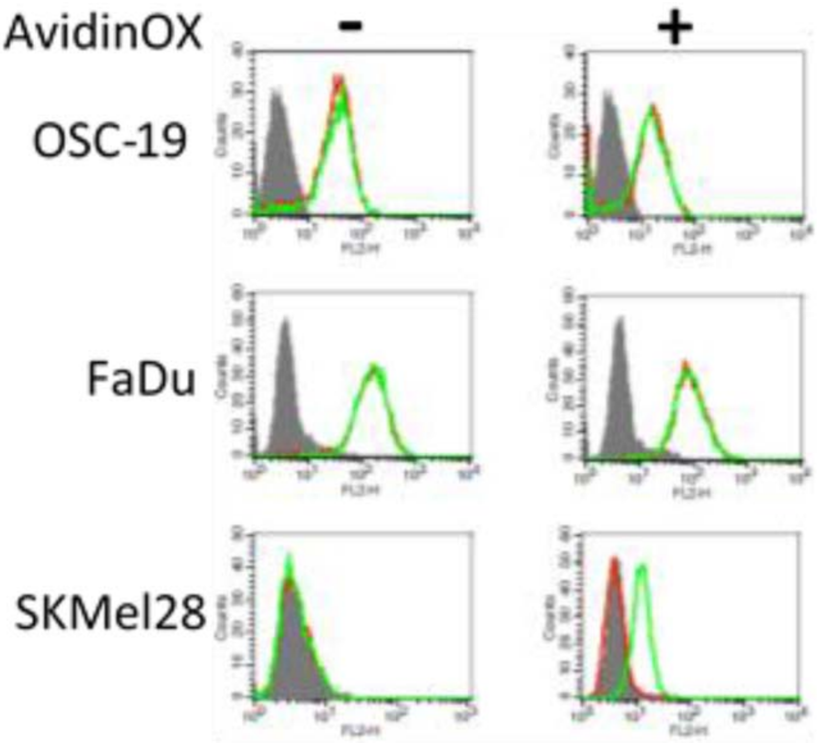

Supplementary Figure S1: Biotinylation does not affect Cetuximab specificity. Cytofluorimetry of Cetuximab (red line) and bCet (green line) antibody binding to tumor cells (EGFR$^+$, OSC-19 and FaDu; EGFR$^-$, SKMel28) with and without AvidinOX pre-conjugation. Antibody binding detected by phycoerythrin-conjugated mouse anti-human Ig. Grey peak, cells without primary antibody.

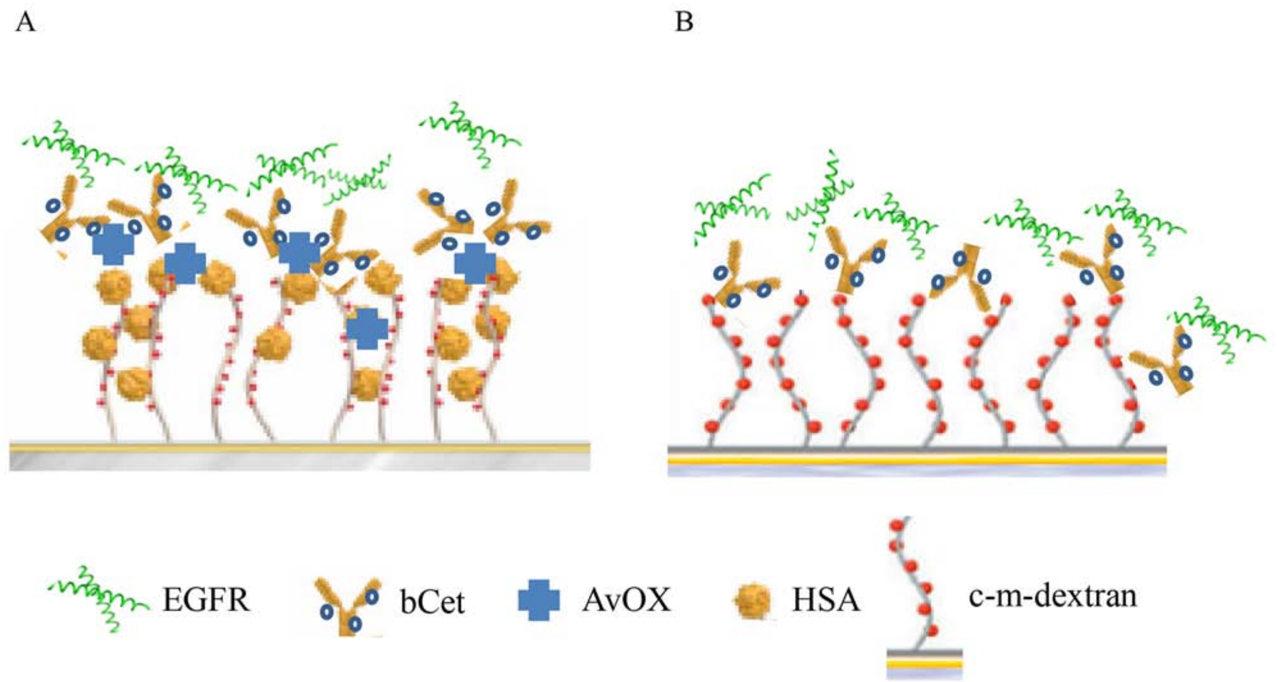

		K_{on} ± SE ($M^{-1}s^{-1}$)	K_{off} ± SE (s^{-1})	K_D (M)
A	HSA-AvOX-bCet	5.20E+04±*8.9E+02*	6.18E-04±*6.6E-05*	1.19E-08
B	bCet	4.79E+04±*1.9E+03*	1.00E-05±*1.2E-06*	2.09E-10

Supplementary Figure S2: SPR evaluation of EGFR interaction with bCet or AvidinOX-bound bCet. One flow cell (panel A) was prepared by immobilizing on carboxyl methyl dextran matrix human serum albumin (HSA) via amino coupling to create a protein layer. Afterwards, AvidinOX was injected to bind HSA via Schiff's bases followed by 100 mM glycine for quenching unreacted groups. Finally, bCet was injected to allow binding to AvidinOX. All interactions proved to be not reversible since regeneration pulses (100 mM NaOH) failed to bring the signal to baseline level. A second flow cell was generated immobilizing bCet via direct amino coupling (panel B). The interaction of recombinant EGFR with bCet in the two conditions was evaluated injecting EGFR in the 200–12.5 nM concentration range using HBS-N as running buffer. In the table are reported the association and dissociation kinetic constants evaluated by BIA Evaluation software version 3.2, and K_D calculated as the ratio of the two constants.

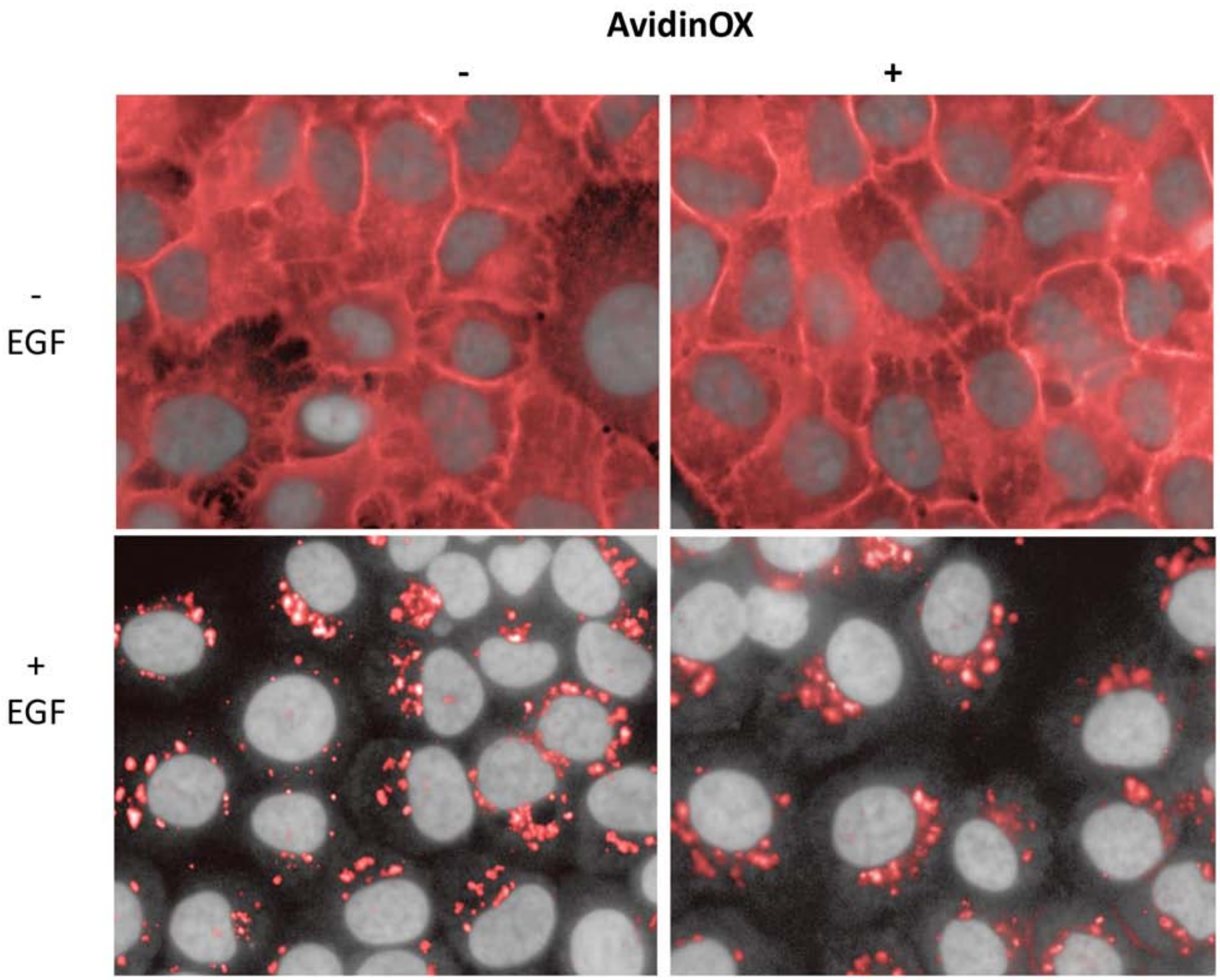

Supplementary Figure S3: EGF-induced EGFR endocytosis in FaDu cells is not affected by AvidinOX conjugation. FaDu cells, with or without AvidinOX conjugation, were incubated 30 minutes with EGF. After washing, the cells were fixed and stained for the detection of EGFR by using AF555-labeled anti-EGFR antibody (red). Draq5-stained nuclei and cytoplasm in grey. Fluorescence images acquired by High Content Screening (HCS) Operetta. Each picture is representative of at least 5 fields of triplicate wells. Magnification 60X.

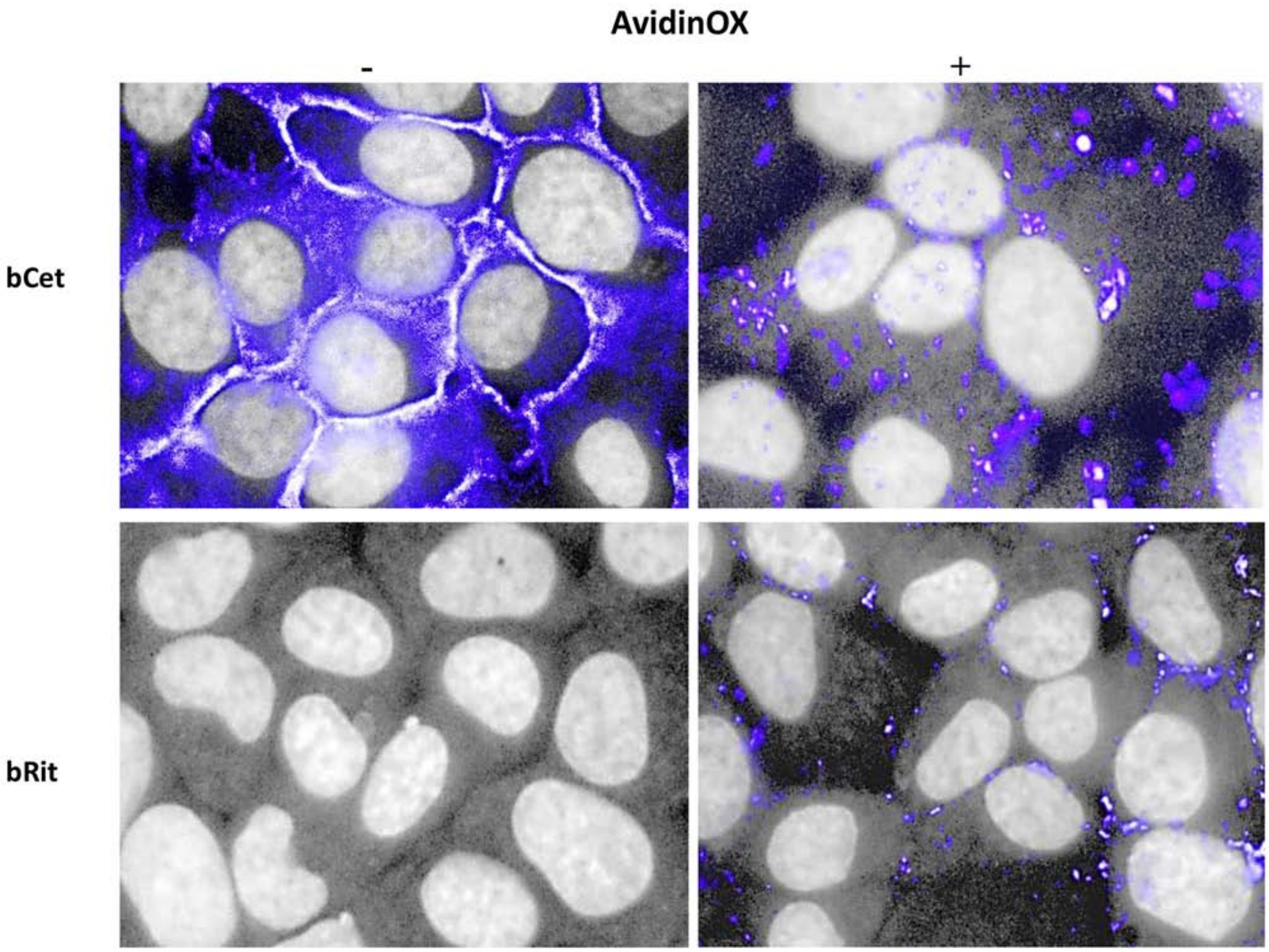

Supplementary Figure S4: bCet internalization is prevented by AvidinOX. FaDu cells, with or without AvidinOX conjugation, were incubated 30 minutes with 5 µg/mL CF488-labeled bCet or bRit (blue). Draq5-stained nuclei and cytoplasm in grey. Fluorescence images acquired by High Content Screening (HCS) Operetta. Each picture is representative of at least 5 fields of triplicate wells. Magnification 60X.

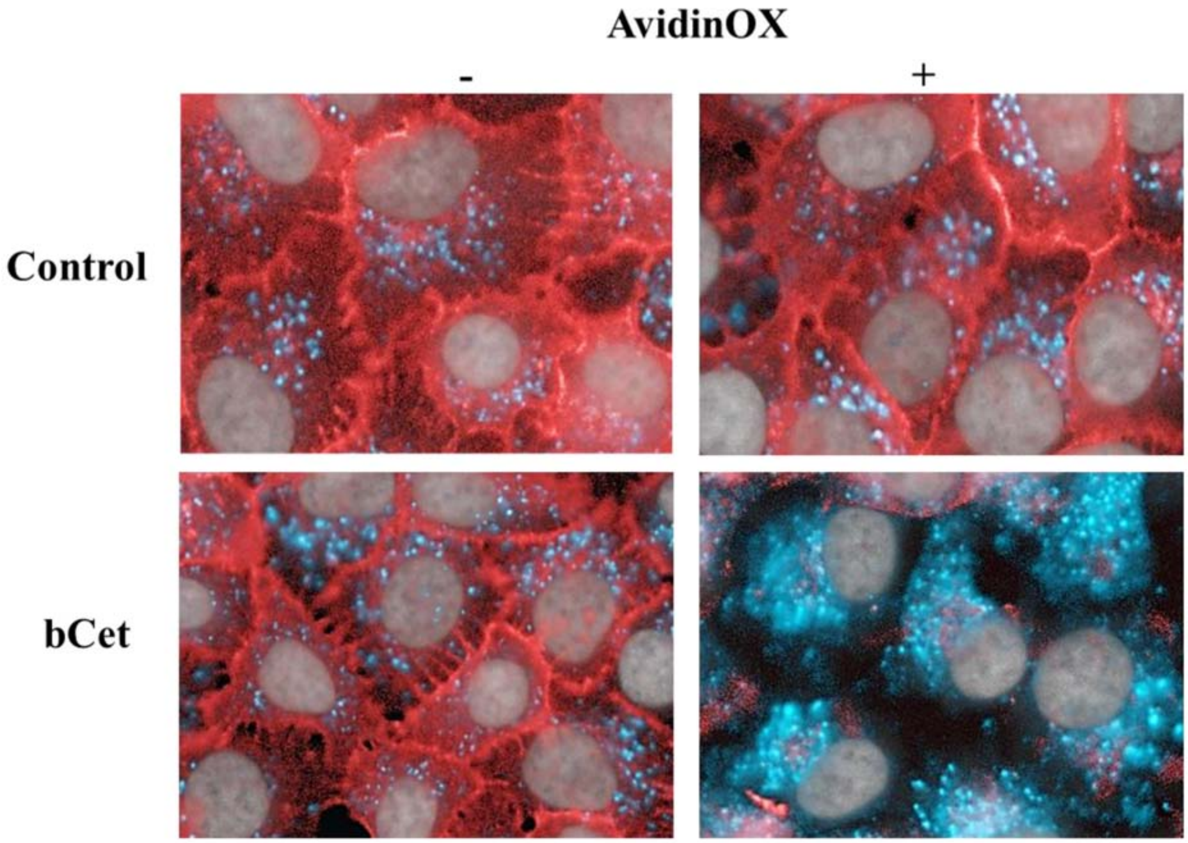

Supplementary Figure S5: AvidinOX-anchored bCet induces lysosomal degradation of EGFR. Fluorescence imaging by High Content Screening (HCS) Operetta of FaDu cells incubated 30 minutes with medium (Control) or 5 µg/mL bCet, with or without AvidinOX. After 24-hour cultivation, the cells were washed, fixed and stained for the detection of EGFR by AF555-labeled anti-EGFR Mab (D38B1) (red). Draq5 dye staining of nucleus and cytoplasm (gray). Staining of lysosomes by anti-LAMP1 antibody (light blue). Pinkish color is the merge of red and blue indicating co-localization of EGFR and lysosomes. All panels: representative picture of at least 5 fields of triplicate wells. Magnification 60X.

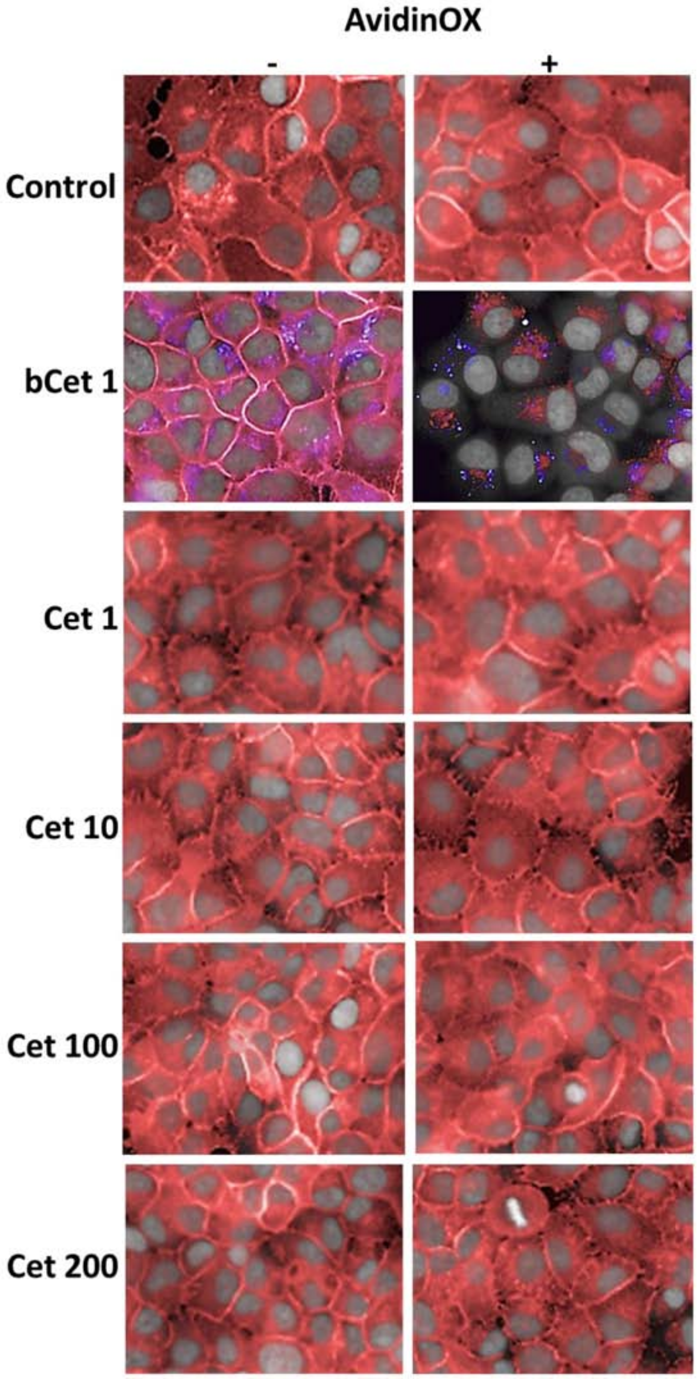

Supplementary Figure S6A: High concentration of Cetuximab does not induce disappearance of EGFR with or without AvidinOX. Fluorescence imaging by High Content Screening (HCS) Operetta of FaDu cells, with and without AvidinOX conjugation and 30 minute incubation with 1 µg/mL CF488-labeled bCet (blue) or Cet at the indicated concentrations (µg/mL). After 24-hour cultivation, cells were washed, fixed and stained for the detection of EGFR by AF555-labeled anti-EGFR Mab (red). Draq5 dye staining of nucleus and cytoplasm (grey). Violet is the result of the blue and red dye co-localization. All panels: representative picture of at least 5 fields of triplicate wells. Magnification 60X.

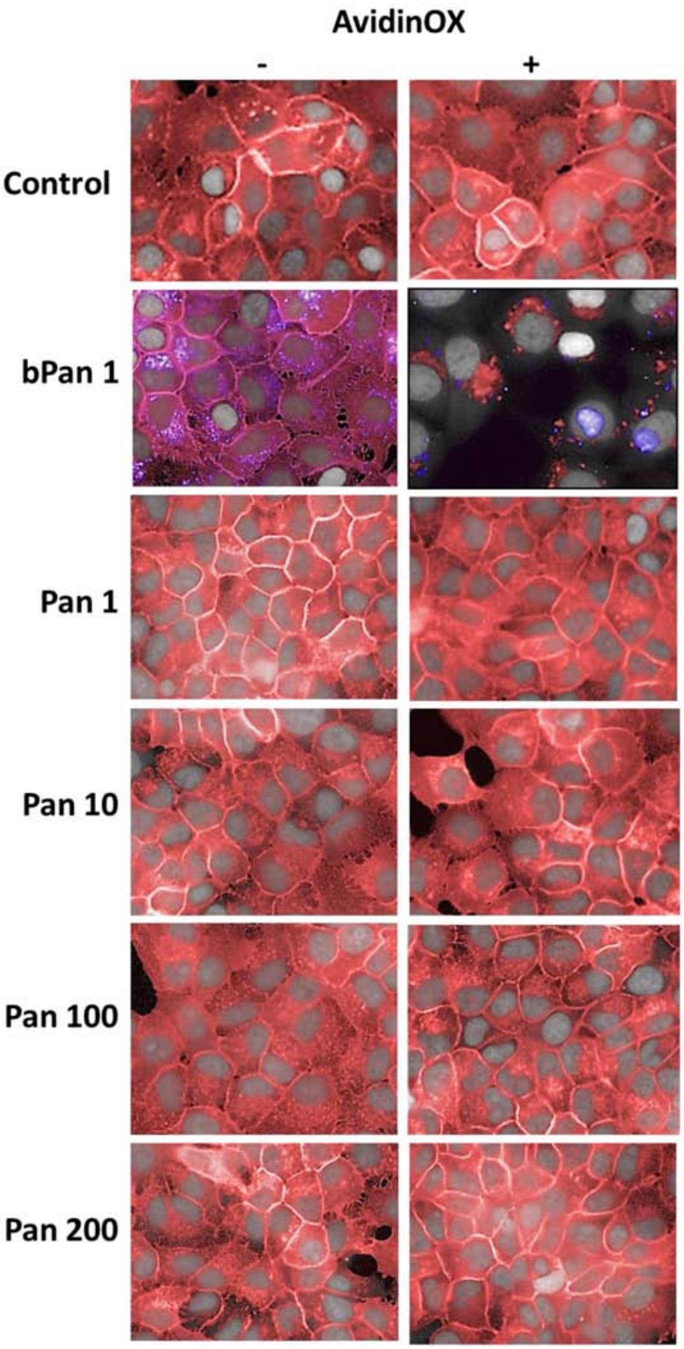

Supplementary Figure S6B (*Continued*): High concentration of Panitumumab does not induce disappearance of EGFR with or without AvidinOX. Fluorescence imaging by High Content Screening (HCS) Operetta of FaDu cells, with and without AvidinOX conjugation and 30 minute incubation with 1 µg/mL CF488-labeled bPan (blue) or Pan at the indicated concentrations (µg/mL). After 24-hour cultivation, cells were washed, fixed and stained for the detection of EGFR by AF555-labeled anti-EGFR Mab (red). Draq5 dye staining of nucleus and cytoplasm (grey). Violet is the result of the blue and red dye co-localization. All panels: representative picture of at least 5 fields of triplicate wells. Magnification 60X.

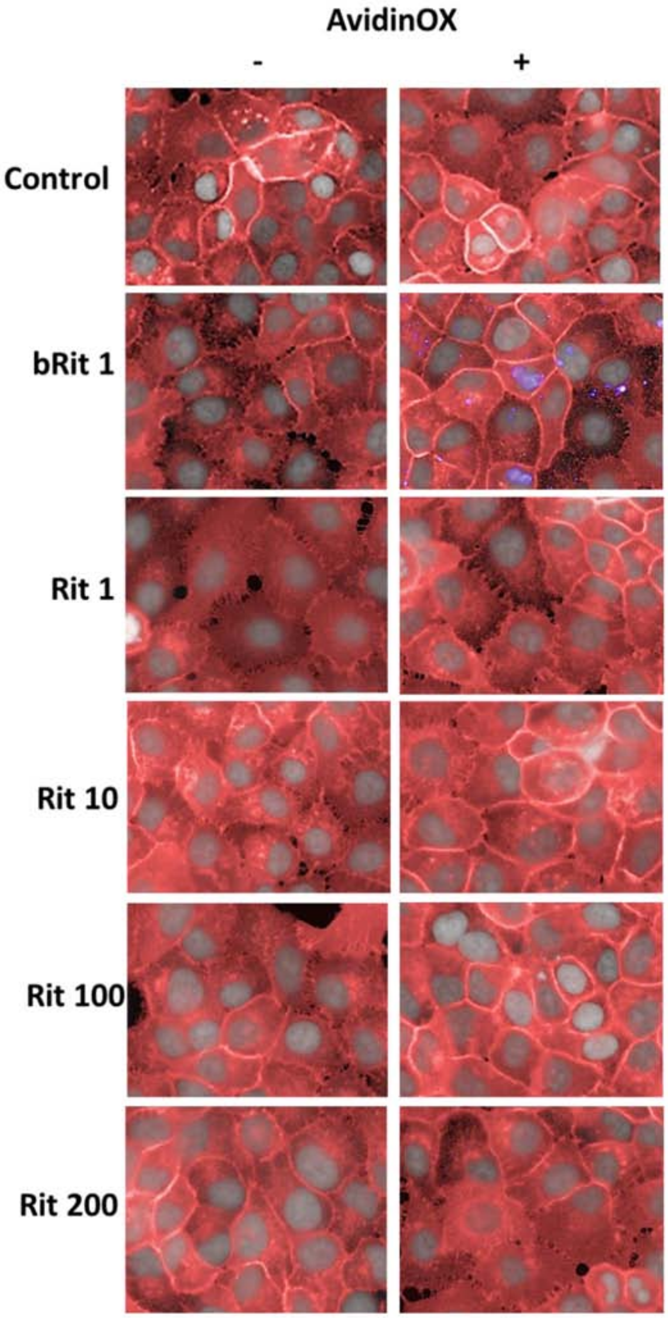

Supplementary Figure S6C (*Continued*): High concentration of Rituximab does not induce disappearance of EGFR with or without AvidinOX. Fluorescence imaging by High Content Screening (HCS) Operetta of FaDu cells, with and without AvidinOX conjugation and 30 minute incubation with 1 µg/mL CF488-labeled bRit (blue) or Rit at the indicated concentrations (µg/mL). After 24-hour cultivation, cells were washed, fixed and stained for the detection of EGFR by AF555-labeled anti-EGFR Mab (red). Draq5 dye staining of nucleus and cytoplasm (grey). All panels: representative picture of at least 5 fields of triplicate wells. Magnification 60X.

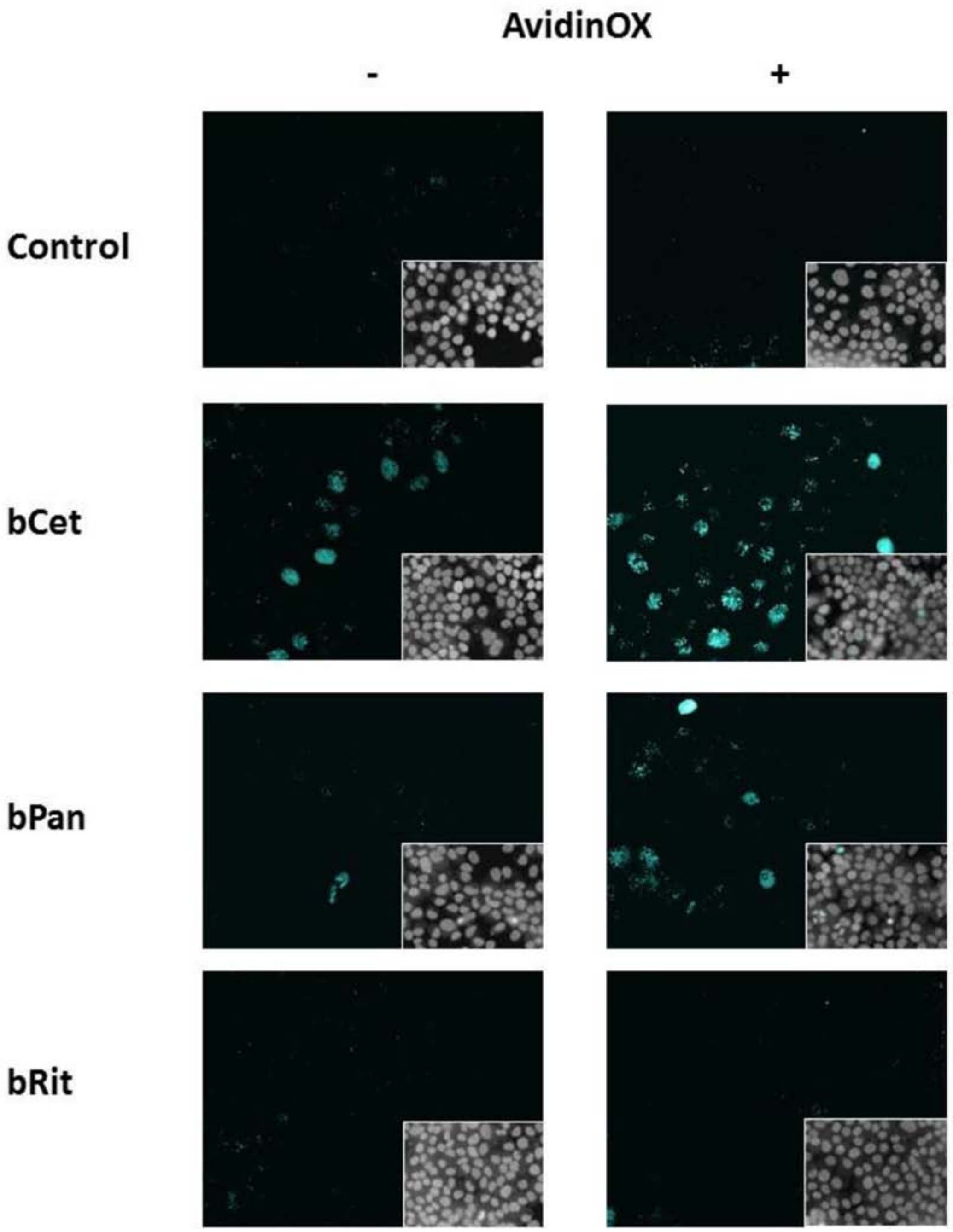

Supplementary Figure S7: P21^{Cip1} is up-regulated by AvidinOX-anchored bCet and bPan but not bRit. FaDu cells, with or without AvidinOX conjugation, were incubated 1 hour with medium (Control) or 5 µg/mL bCet, bPan or bRit and, after washing, cultivated 48 hours with EGF induction the last 30 minutes. Cells were then washed, fixed and stained for the detection of P21^{Cip1} (light blue). Draq5-stained nuclei and cytoplasm in grey (insets). Fluorescence images acquired by High Content Screening (HCS) Operetta. Each picture is representative of at least 5 fields of triplicate wells. Magnification 60X.

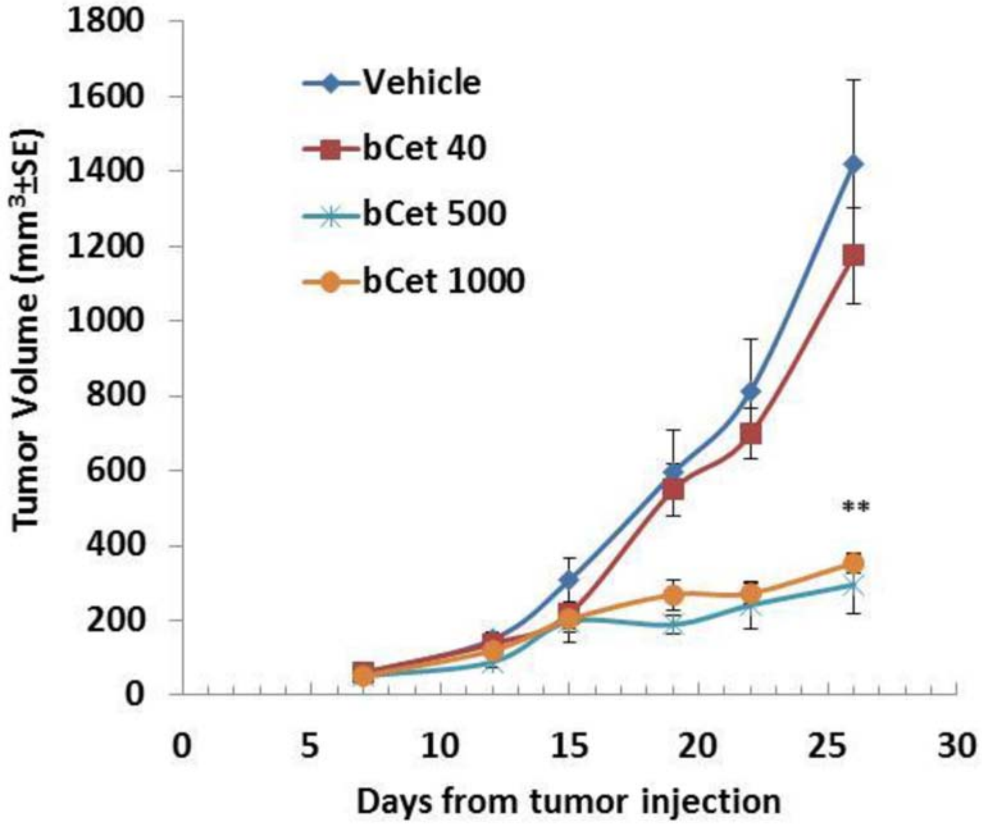

Supplementary Figure S8: Tumor growth inhibition by intraperitoneally administered bCet. Growth curves of FaDu tumor xenografts in nude mice (8/group) upon intraperitoneal administration of 100 µL vehicle or bCet at the indicated doses (µg), once a week for three weeks, starting 7 days after tumor implantation. Tumor volume estimated by digital Vernier Caliper. Tumor masses at start of treatment 40–50 mg. Data are mean ± SE. ***p* < 0.01 vs vehicle-treated group, Mann-Whitney's U test.
